# Host choice in a bivoltine bee: how sensory constraints shape innate foraging behaviors

**DOI:** 10.1186/s12898-016-0074-z

**Published:** 2016-04-11

**Authors:** Paulo Milet-Pinheiro, Kerstin Herz, Stefan Dötterl, Manfred Ayasse

**Affiliations:** Institute of Evolutionary Ecology and Conservation Genomics, University of Ulm, Helmholtzstraße 10-1, 89081 Ulm, Germany; Department of Ecology and Evolution, University of Salzburg, Hellbrunnerstrasse 34, 5020 Salzburg, Austria; Departamento de Química Fundamental, Universidade Federal de Pernambuco, Av. Prof. Moraes Rego, s/n, Recife, 50670-901 Brazil

**Keywords:** *Andrena bicolor*, *Campanula*, Olfactory and visual cues, Oligolecty, Polylecty, Seasonal polyphenism

## Abstract

**Background:**

Many insects have multiple generations per year and cohorts emerging in different seasons may evolve their own phenotypes if they are subjected to different selection regimes. The bivoltine bee *Andrena bicolor* is reported to be polylectic and oligolectic (on *Campanula*) in the spring and summer generations, respectively. Neurological constraints are assumed to govern pollen diet in bees. However, evidence comes predominantly from studies with oligolectic bees. We have investigated how sensory constraints influence the innate foraging behavior of *A. bicolor* and have tested whether bees of different generations evolved behavioral and sensory polyphenism to cope better with the host flowers available in nature when they are active.

**Results:**

Behavioral and sensory polyphenisms were tested in choice assays and electroantennographic analyses, respectively. In the bioassays, we found that females of both generations (1) displayed a similar innate relative reliance on visual and olfactory floral cues irrespective of the host plants tested; (2) did not prefer floral cues of *Campanula* to those of *Taraxacum* (or vice versa) and (3) did not display an innate preference for yellow and lilac colors. In the electroantennographic analyses, we found that bees of both generations responded to the same set of compounds.

**Conclusion:**

Overall, we did not detect seasonal polyphenism in any trait examined. The finding that bees of both generations are not sensory constrained to visit a specific host flower, which is in strict contrast to results from studies with oligolectic bees, suggest that also bees of the second generation have a flexibility in innate foraging behavior and that this is an adaptive trait in *A. bicolor*. We discuss the significance of our findings in context of the natural history of *A. bicolor* and in the broader context of host-range evolution in bees.

**Electronic supplementary material:**

The online version of this article (doi:10.1186/s12898-016-0074-z) contains supplementary material, which is available to authorized users.

## Background

Bees visit flowers mainly to collect nectar and pollen. These floral rewards are essential for both their own nutritional requirements and brood provision [1]. The spectrum of plants visited for pollen collection varies greatly from one species to another. Some bees restrict pollen gathering to a few (single) species within a genus or family (oligolecty), whereas others collect pollen from various species of distinct families (polylecty) [2, 3].

The evolution of pollen preference in bees has long puzzled scientists but has only recently received special attention. In contrast to previous long-held assumptions (see for example, [4–6]), recent studies show that many polylectic lineages are derived from oligolectic ancestors [7–10]. Whereas the basal state of oligolecty is now well acknowledged, the ecological and physiological aspects governing host choice in bees remain poorly investigated. In bee-plant interactions, pollen plays a paradoxical role; it is, on the one hand, the male gametophyte of plants but, on the other hand, the food of future pollinators (i.e. bee larvae). Consequently, in addition to being pollinators, bees can be seen as herbivores and plants might be under selective pressure to reduce pollen harvesting by animals [11]. Recently, plants have been suggested to have evolved chemical defensive properties (i.e. toxic pollen) to avoid excessive pollen consumption [12, 13]. These defensive properties have to be overcome by bees and are assumed to influence pollen diet. Furthermore, nutritional content (e.g. protein, amino acids, lipids, etc.) and the digestibility of pollen vary immensely among the different taxa [14] and, thus, the digestion of various types of pollen might be challenging for bees [15]. Accordingly, evidence is available that the ability of bee larvae to digest and develop on different pollen types varies considerably among species and this holds true for both oligolectic [12, 16, 17] and polylectic [13, 18, 19] representatives. Together, these findings show that pollen is not an easy-to-use resource and that physiological adaptations are necessary for efficient pollen digestion [10, 12, 13, 20].

In addition to physiological constraints, neurological adaptations are assumed to govern host range in bees [12, 13]. However, the exact mechanisms by which neurological adaptations govern host-flower preference remain elusive. Linsley [21] was the first to speculate that the tendency of newly-emerged oligolectic bees to collect pollen only on host flowers used by the previous generation arises from their experience with pollen aroma during the larval stage (i.e. imprinting or conditioning). Although intensively discussed in the literature, this hypothesis has been tested only in three solitary species whose pollen preferences vary considerably (Tepedino, cited in [22–24]). The results of these studies suggest that host-flower preference is genetically controlled. Dobson et al. [24], for example, have found that adults of the broadly polylectic *Osmia bicornis* (Megachilidae), which had been reared during the larval stage on pollen loads of either *Brassica napus* (Brassicaceae) or *Onobrychis viciifolia* (Fabaceae), showed no clear preference for these two hosts when each was offered them together with seven alternative host plants. In *Megachile rotundata* (Megachilidae), a polylectic bee with a more restricted pollen diet, adults selected their preferred host, *Medicago sativa* (Fabaceae), even if they had been reared on a pure pollen diet of *Daucus* (Apiaceae) (Tepedino, cited in [22]). Finally, Praz et al. [23] have found that females of *Heriades truncorum* (Megachilidae) restrict pollen gathering to their hosts (Asteraceae), irrespective of the pollen diet on which they had been reared as larvae, and have suggested that the innate foraging behavior of oligolectic bees is constrained by genetically based neurological adaptations (e.g. vision and olfaction) that drive their flight towards host flowers. Obviously, the extent to which these findings can be generalized to other bee species remains to be established.

Studies investigating the role of visual and olfactory floral cues in the innate foraging behavior of solitary bees are scarce and have focused mainly on oligolectic species. Nevertheless, they provide interesting insights into the way that neurological adaptations can restrict host range in bees. The general tendency emerging from these studies suggests that oligolectic bees innately prefer visual and olfactory floral cues of host plants over those of non-host plants [25–28], thereby implying that oligolectic bees are neurologically adapted to detect some cues that are characteristic for host flowers. In terms of visual cues, for example, some oligolectic species have been shown to display an innate preference for the color of host flowers [26, 28]. According to these authors, color might act as a filter that drives foraging flights of bees towards potential host flowers but, given its unspecific nature (i.e. flowers of species belonging to very distinct taxa might share the same color), color alone would not be a reliable cue for host recognition. Instead, they suggest that floral scents, which are assumed to have an infinite diversity [29, 30], provide a reliable signature for host flowers. Indeed, several oligolectic bees have been shown to rely innately on a single or a few host-typifying floral scent compounds in order to recognize host flowers [25, 31–35]. This might prevent oligolectic bees foraging for pollen on non-host plants that might not be digestible by their larvae. Further studies focusing on the innate reliance on visual and olfactory cues by bees with a different degree of pollen preference would help in understanding the way that sensory adaptations shape the evolution of host range in bees as a whole.

Unlike oligolectic bees, which normally have a very short flight period that is synchronized with the flowering of their host plants, some polylectic bees exhibit long flight activity and present two (bivoltinism) or more (multivoltinism) generations per year [1, 36]. Multivoltine insects (including bees) are assumed to be under distinct selective pressures depending on the biotic (e.g. food availability, predators) and abiotic (e.g. temperature, day length) conditions of the environment in which each generation appears (reviewed by [37–39]). Thus, individuals of distinct generations might evolve their own phenotypes to cope better with specific conditions typical to their surrounding environment, a phenomenon known as polyphenism (i.e. distinct phenotypes produced by the same genotype) [39]. Indeed, many insects show seasonal polyphenism that can influence a variety of traits, morphology and color being the most well investigated [38, 40]. Naturally, the same differences in selection pressures that cause seasonal polyphenism in morphology and color are also likely to cause behavioral and neurological polyphenisms. Evidence of seasonal behavioral and neurological polyphenisms exists for insects such as butterflies (measured as mating propensity; [41, 42]) and locusts [measured as brain size; [43], respectively, but have never been investigated in any respect (e.g. host preference, color preference and olfactory receptors) in bivoltine bees.

The ability of bees to detect a given floral scent compound is often assessed by gas chromatography coupled to electroantenographic detection (GC-EAD), an analytical technique in which an insect antenna is used as a parallel detector for compounds separated on a GC column to identify subsets of complex odor blends that are physiologically active and likely show biological activity [44]. Assuming that first- and second-generation individuals of bivoltine bees are under distinct selective pressure exerted by the different host flowers, and thus different scents, available in each season, we might expect that individuals of each generation would sensory evolve to detect better their respective host plants. Under this perspective, it seems reasonable to speculate that the antennae of first-generation bees respond to a higher number of compounds released by plants flowering in the spring than antennae of first-generation bees and vice versa.

**Fig. 1:**
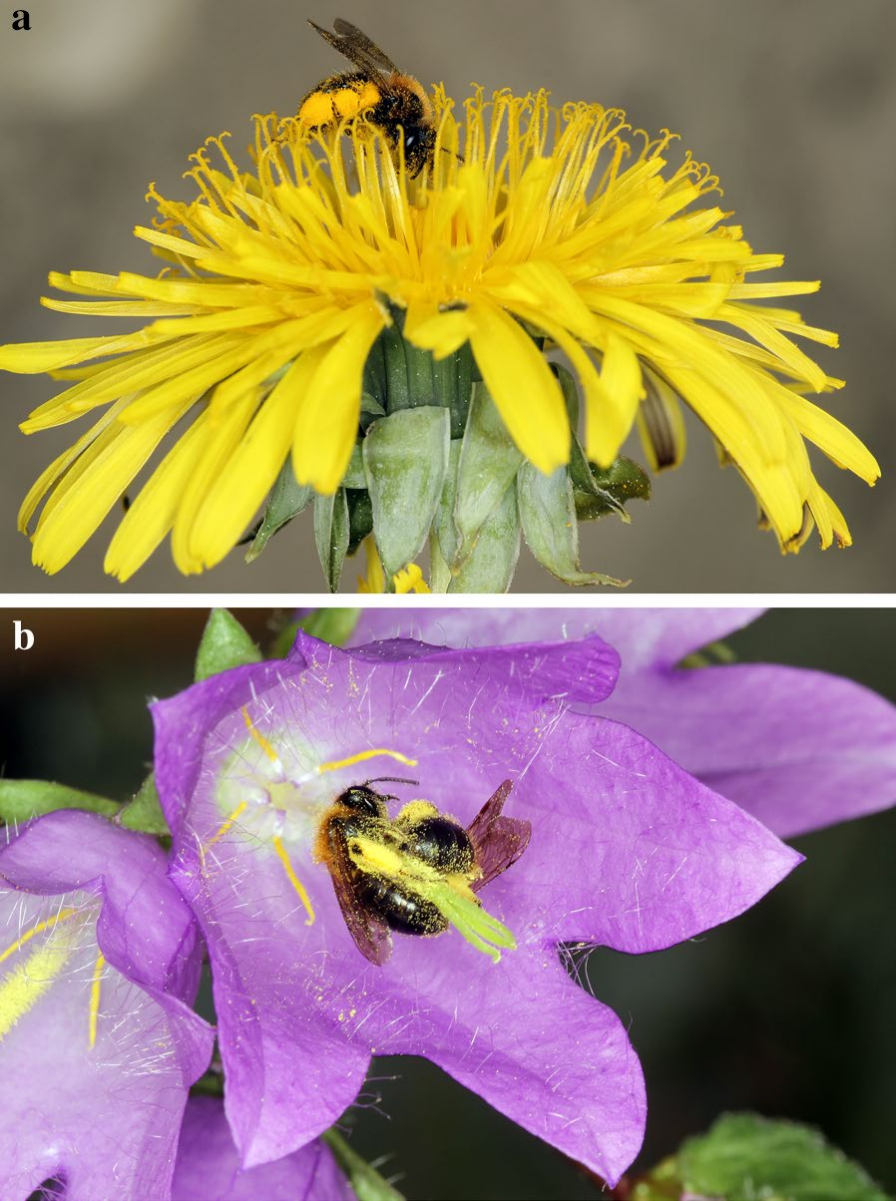
*Andrena bicolor* and host plants used in the present study. Females of the first and second generation on flowers of *Taraxacum officinale* (**a**) and *Campanula trachelium* (**b**), respectively (Photographs: P. Milet-Pinheiro)

*Andrena bicolor* (Fabricus 1775) (Andrenidae) is a European solitary bivoltine species. Adults of the first generation are active between March and May and those of the second generation between June and August. Whereas the first generation is assumed to be polylectic, the second has a strong preference for flowers of *Campanula* [45, 46], (Westrich, pers. comm.). In the present study, we have investigated the innate responses of *A. bicolor* females to visual and olfactory floral cues of two common host plants, namely *Taraxacum officinale* (Fig. 1a) and *Campanula trachelium* (Fig. 1b), whose blooming peaks coincide with the flight activity of the first and second generation, respectively. We have hypothesized that bees of the second generation have evolved behavioral and sensory adaptations to cope better with flowers of *Campanula*, whereas bees of the first generation have evolved adaptations to cope with a broader spectrum of host flowers. In order to test this possible seasonal polyphenism, we have integrated behavioral and electrophysiological methods and addressed the following questions:

1. Does the relative reliance on visual and olfactory floral cues of host plants differ between bees of the first and second generation?
2. Do bees of the first and second generation prefer the floral cues (i.e. visual and olfactory) of their common pollen hosts, *Taraxacum* and *Campanula*, respectively?
3. Do bees of the two generations have different color preferences?
4. Are there differences in antennal responses to volatiles of *Taraxacum* and *Campanula* between bees of the two generations?
5. Does the preference of adults for floral cues of either *Taraxacum* or *Campanula* (if any) reflect the pollen diet provided during the larval stage?

##  Methods

### The host plants

*Taraxacum officinale* F.H Wigg (Asteraceae) is a widely distributed herb capable of growing under diverse environmental conditions. The yellow flowers provide nectar and pollen and are attractive to several floral visitors, mainly bees [47]. The plant blooms from April to October (Schmeil-Fitschen [48]) and is reported as a pollen source for the first generation bees of *A. bicolor* [36, 45].

*Campanula trachelium* L. (Campanulaceae) has hermaphroditic violet flowers and blooms between July and September (Schmeil-Fitschen [48]). It is protandrous and pollen is secondarily presented on the style [49]. For experiments with bees of the first generation (between late March and early April), *C. trachelium* plants were cultivated in pots in the plant beds of the Botanical Garden of the University of Ulm. During late January, plants were placed in the greenhouses where they were subjected to controlled light (16 h day length) and temperature (10–12 °C) conditions to stimulate flowering during late March. Plants were vigorous and produced several flowers.

### Establishment of the bee population in a flight cage

For bioassays, *A. bicolor* bees were reared in a flight cage at the Botanical Garden of the University of Ulm. The flight cage consisted of a steel frame (7 × 3.5 × 2.2 m) that was covered with a fine mesh (stitch density of 1 mm × 0.5 mm) and whose base was buried into the soil to a depth of 0.5 m.

For the establishment of the population, females of the second generation were caught in the Botanical Garden of Ulm and at wild vegetations in the “Schwäbische Alb” at the vicinities of Ulm while foraging on flowers in the summer of 2012. Individuals were exclusively found on *Campanula* flowers, even if we have searched for bees on flowers of other species. The bees were then released into the cage in which flowering plants of *C. trachelium* had been placed. After a few days, bees collected pollen and built nests in the ground.

### Flower-naive bees

To test the innate responses of *A. bicolor* to floral cues of *T. officinale* and *C. trachelium*, we performed a series of two-choice bioassays (see details below) with flower-naive female bees of the first and second generation that had hatched from nests inside the cage. Flower-naive bees were defined as those that had had no previous contact with any kind of flower as adults. During the phase in which the bioassays were performed, bees were provided only with sugar water (30 % fructose and glucose 1:1) presented in black sponge feeders. In the flight cage, bees of the first generation (97 males and 76 females) emerged in early April 2013, whereas those of the second generation (100 males and 87 females) emerged in early June 2013. After completion of the bioassays with flower-naive bees of the first and second generations, we introduced plants of *T. officinale* and *C. trachelium* into the flight cage, respectively. The bees promptly started to gather pollen and nectar on flowers and to build nests in the ground. By doing this, we forced bees of the first and second generation to provide offspring exclusively with pollen of *T. officinale* and *C. trachelium*, respectively.

### General design of bioassays

#### Experimental cylinders

**Fig. 2:**
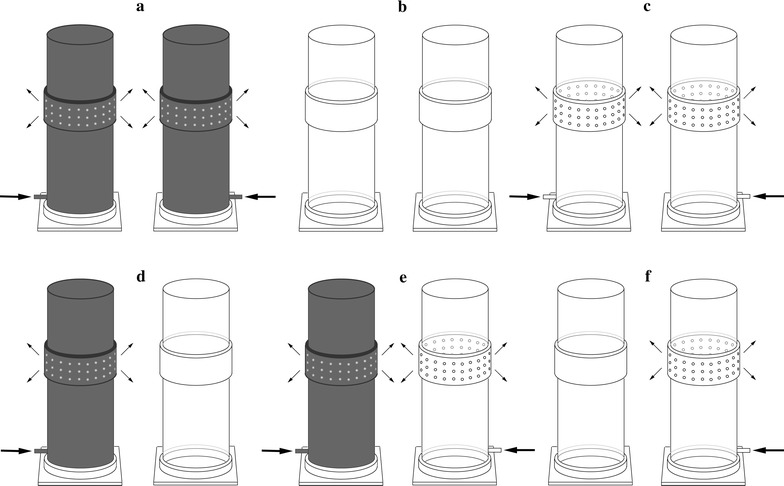
Overview of the cylinders and experimental setup used for bioassays. Two identical cylinders each were used to test the decoupled olfactory (**a**) and visual cues (**b**) or coupled cues (**c**), either of *Taraxacum* or *Campanula*, vs. respective negative controls (*empty cylinders*), and to test olfactory, visual or olfactory + visual cues of *Taraxacum* vs. those of *Campanula*. Two different types of cylinders each were used to test olfactory vs. visual cues (**d**) and the coupled cues vs. either olfactory (**e**) or visual (**f**) cues of either *T. officinale* or *C. trachelium*. *Arrows* indicate air flow (1 l min^−1^), which was generated by a membrane pump

**Fig. 3:**
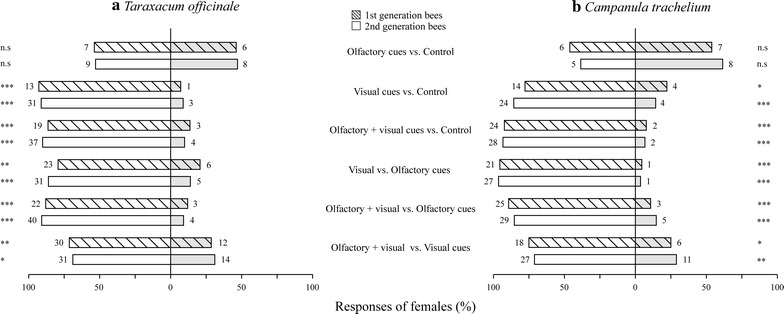
Relative importance of floral cues of *Taraxacum officinale* (**a**) and *Campanula trachelium* (**b**) to *Andrena bicolor*. Responses (approaches + landings) of flower-naive females of the first and second generation to olfactory and visual cues (alone or combined) of host flowers or to empty controls in dual-choice bioassays. *Numbers given at the right and left side of the bars* indicate the absolute number of responding bees. Differences in responses for each dual-choice bioassay were assessed by an exact binominal test, *P* > 0.05 (n.s.), *P* < 0.05 (*), *P* < 0.01 (**), *P* < 0.001 (***)

The attractiveness of decoupled and combined olfactory and visual floral cues of *T. officinale* and *C. trachelium* to *A. bicolor* females was tested with three kinds of cylinders, namely (1) decoupled olfactory cues: grey cylinders with 60 small holes (diameter 0.2 cm) (Fig. 2a); (2) decoupled visual cues: transparent solid cylinders, without holes (Fig. 2b); (3) coupled olfactory and visual cues: transparent cylinders with 60 small holes (Fig. 2c). Air containing floral scents from the enclosed inflorescences in cylinders (1) and (3) was blown out of the holes by a membrane pump (G12/01 EB; Rietschle Thomas, Puchheim, Germany) at a flow rate of 1 l min^−1^. All cylinders had dimensions of 39 cm height and 10 cm diameter. Grey cylinders were made from PVC and transparent cylinders from Plexiglas^®^. Plexiglas was used because of its ultraviolet (UV) transparency. Plant samples, which consisted of 5–8 inflorescences with pedicel lengths of 15 cm for *T. officinale* and one inflorescence with 5–8 flowers and 20 cm pedicel length for *C. trachelium*, were covered with either grey or transparent cylinders (see details below). For all bioassays, the same number of *Campanula* and *Taraxacum* flowers was used.

#### Relative importance of visual and olfactory cues for host location

**Fig. 4:**
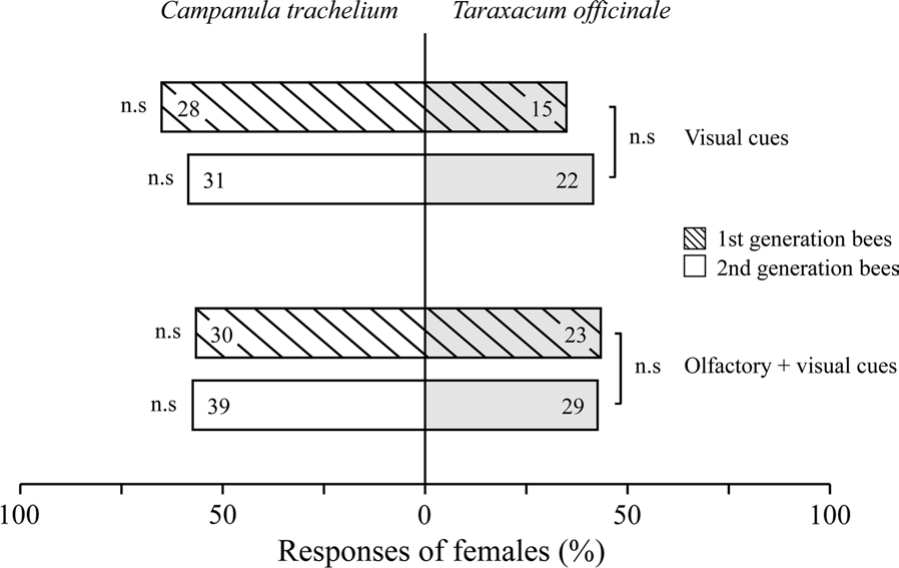
Bioassays comparing the attractiveness of floral cues of *Taraxacum officinale* vs. those of *Campanula trachelium* to *Andrena bicolor*. Responses (approaches + landings) of flower-naive females of the first and second generation to visual cues alone or combined with olfactory cues of *T. officinale* compared with *C. trachelium* in dual-choice bioassays. *Numbers within bars* indicate the absolute number of responding bees. The difference in responses in each dual-choice bioassay was assessed by an exact binominal test and is shown at the *left*-*hand side of the bars*, *P* > 0.05 (n.s). Results of the Fisher’s exact tests used to compare the responses of flower-naive females of the first and second generation to each cue are shown at the *right*-*hand side of the bars*, *P* > 0.05 (n.s)

**Fig. 5:**
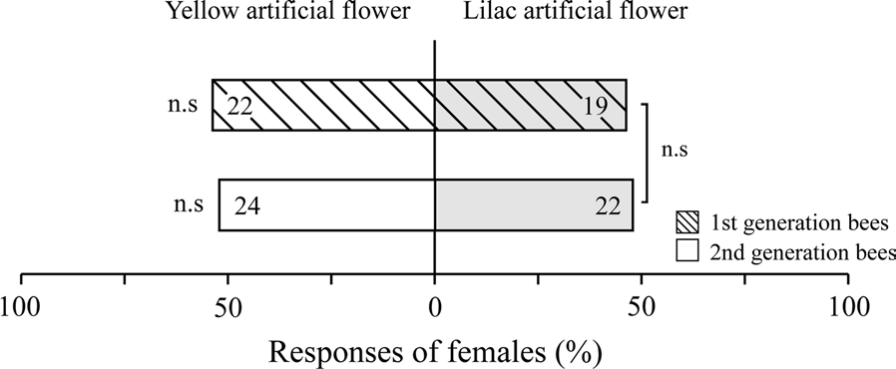
Responses (approaches + landings) of flower-naive females of *Andrena bicolor* of the first and second generation to yellow and lilac artificial flowers in dual-choice bioassays. *Numbers within bars* indicate the absolute number of responding bees. The difference in responses for each dual-choice bioassay was assessed by an exact binominal test and is shown at the *left*-*hand side of the bars*, *P* > 0.05 (n.s). Results of the Fisher’s exact test used to compare the responses of flower-naive females of the first and second generation to the two artificial flowers is shown at the *right*-*hand side of the bars*, *P* > 0.05 (n.s)

**Fig. 6:**
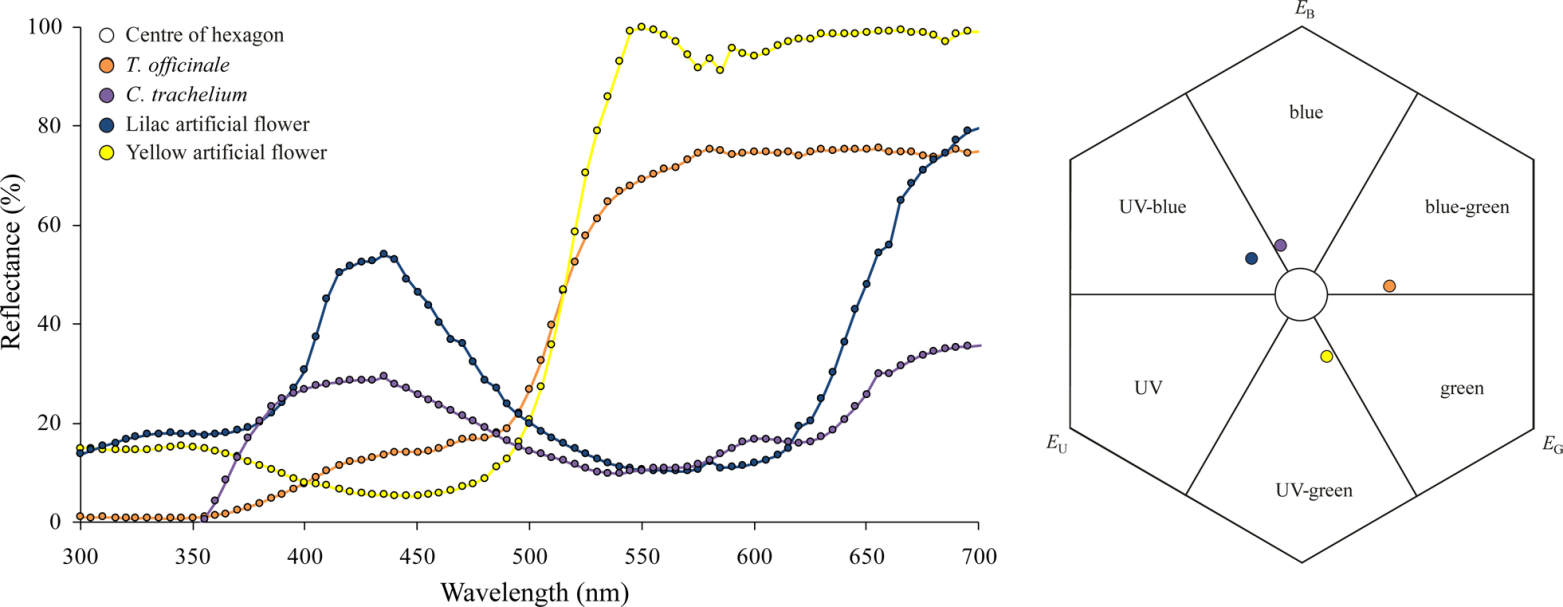
Color reflectance and bee color space. Spectral reflectance curves of the flowers of *C. trachelium* and *T. officinale* and of the yellow and lilac artificial flowers (*n* = 3 measurements for each) and corresponding color loci plotted in a *hexagon color space* against a standard green leaf. *EU*, *EB*, *EG* represent the excitation of the UV, blue and green receptor, respectively. The pairwise distances between the color loci and between each color locus and the uncolored point (centre of the hexagon) are given in Additional file 3

To test whether the relative reliance on visual and olfactory floral cues differed in females of the two generations, we conducted six dual-choice bioassays for each host plant. The bioassays were carried out in the following order: (1) olfactory cues vs. empty control (Fig. 2a), (2) visual cues vs. empty control (Fig. 2b), (3) olfactory + visual cues vs. empty control (Fig. 2c), (4) olfactory vs. visual cues (Fig. 2d), (5) olfactory + visual vs. olfactory cues (Fig. 2e) and (6) olfactory + visual vs. visual cues (Fig. 2f).

#### Attractiveness of *T. officinale* vs. *C. trachelium*

To establish whether (1) *A. bicolor* preferred the floral cues of *T. officinale* to those of *C. trachelium* (or vice versa) and (2) whether an eventual preference differed between both generations, three dual-choice bioassays were performed. In the bioassays, females of both generations were offered a choice of floral cues of *T. officinale* vs. *C. trachelium* in the following order: (1) olfactory cues (Fig. 2a), (2) visual cues (Fig. 2b) and (3) olfactory + visual cues (Fig. 2c).

#### Bioassay protocol

The bioassays were conducted on sunny days between 10:00 a.m and 2:00 p.m (when bees were most active). Each bioassay lasted 30 min; the position of the paired cylinders, which were placed 1 m apart, was exchanged after 15 min. Responses of the bees were characterized as either (1) approaches: flights toward the cylinder, to a distance closer than 10 cm, without landing or (2) landings: approaches followed by landing on the cylinders. All responding bees were collected, by using nets, after they had responded in order to prevent them from interfering in the attraction of other individuals; we never observed a responding bee being ‘‘followed’’ by another bee. Approaching bees were collected when they flew away from the cylinders and landing bees after they had landed. All responding bees were stored in an icebox until the end of the experiment, at which time they were released back into the flight cage and could participate in subsequent tests. Thus, an individual bee could respond only once in each specific bioassay.

#### Color preference

To test whether *A. bicolor* females displayed an innate preference for a given color and whether color preference differed according to bee generation, dual-choice experiments with artificial flowers were performed. For these experiments, we selected human-lilac and human-yellow colors, because of both their representativeness in nature and their high attractiveness to insects in general [50–52]. Although the colors of the artificial flowers used here did not resemble exactly the colors of flowers of *T. officinale* and *C. trachelium* (see results), they did resemble those of several Asteraceae (Milet-Pinheiro, unpublished data) and Campanulaceae [28]. Lilac and yellow bell-shaped artificial flowers (length 4.5 cm; diameter at the top 2 cm), were fashioned out of construction paper and each artificial flower was fixed at the base on a thin wooden stick (length: 23 cm; diameter: 2 mm). Two groups of artificial flowers (choices) were presented to the bees simultaneously. Each group was composed of three artificial lilac flowers or three yellow flowers. Artificial flowers within a group were arranged in a triangle and 5 cm apart from each other. The two groups of artificial flowers were placed 1 m apart. The general testing procedures were the same as those for the cylinder tests (see bioassay protocol).

### Evaluation of visual and olfactory cues

#### Color measurements and bee color hexagon

To improve our understanding of the significance of color in the innate behavioral responses of *A. bicolor*, we measured reflectance properties of both natural (*n* = 3 for each species) and artificial (*n* = 3 measurements per paper) flowers by using a Varian Cary 5 spectrophotometer equipped with a Praying Mantis accessory (Varian, Inc, Palo Alto, California). The mean reflectance profiles were then converted into color loci of the color hexagon space [53]. The hexagon space is a model of bee color vision applicable to a large number of hymenopteran species and allows an interpretation of the way in which colors are perceived and discriminated by bees (for full details concerning reflectance measurements and bee color hexagon, see Additional file 1).

#### Sampling of floral scent

To obtain samples for electrophysiological investigations (*n* = 1 per species from 50 flowers each), floral volatiles from the flowers of *T. officinale* and *C. trachelium* were collected by using a standard dynamic headspace method (for full details of the procedures applied and material used, see Additional file 1).

#### Electrophysiology

Analyses of gas chromatography coupled with electroantennographic detection (GC/EAD) were performed with *A. bicolor* females (*n* = 5 antennae for each generation) to determine the antennal perception of the bee for compounds in the floral scent bouquet of *T. officinale* and *C. trachelium* [for full details of equipment specifications and configurations and of antennal preparation, Additional file 1).

#### Chemical analyses

To identify the floral volatiles eliciting antennal depolarization in *A. bicolor*, the headspace samples of *T. officinale* and *C. trachelium* were analyzed on a gas chromatograph coupled to a mass spectrometer (GC/MS) (for full details of equipment specifications and configurations and of the elucidation and quantification of compounds, see Additional file 1).

## Statistical analyses

To test for differences in total bee responses (pooled approaches and landings) between the paired treatments in each bioassay, exact binominal tests were performed. The two types of behaviors were pooled because visual cues of flowers of *T. officinale* and *C. trachelium* and a combination of visual and olfactory cues triggered approach and landing responses in similar proportions (Fisher’s exact tests: 0.49 < *P* < 1, see Additional file 2). In the bioassays testing the relative importance of floral cues within a plant species, one-tailed exact binomial tests were used to test the null hypothesis that both visual and olfactory cues attract ≤number of bees than the negative controls (empty cylinders) and that combined cues attract ≤number of bees than the decoupled cues. The one-tailed design was used because it is highly unlikely that visual and olfactory cues of *T. officinale* and *C. trachelium* have repellent properties for *A. bicolor*. For the bioassays testing cues of *T. officinale* vs. those of *C. trachelium*, two-tailed exact binominal tests were used to test the hypothesis that floral cues (alone or in combination) of both species are equally attractive to bees. Fisher’s exact tests were used to test whether bees of the first and second generation displayed distinct preferences for visual and olfactory floral cues (either alone or in combination) of a given host. Binomial and Fisher’s exact tests were calculated by using the spreadsheets provided by http://www.biostathandbook.com/exactgof.html and http://www.biostathandbook.com/fishers.html, respectively (Accessed 10 Nov 2014; see also [54]).

Differences in antennal responses between females of the first and second generation of *A. bicolor* were tested by using an analysis of similarity (ANOSIM). For this purpose, we prepared a table with the EAD responses (presence/absence) of bees of the first and second generation. We then calculated the Sørensen similarity index. This index determines pairwise similarities among the individuals. Based on this similarity matrix, we performed an ANOSIM analysis (10,000 permutations) considering the generation as a factor. ANOSIM yields a test statistic R that is a relative measure of separation among a priori defined groups. It is based on differences of mean ranks among and within groups. An R value of ‘0’ indicates random grouping, whereas a value of ‘1’indicates that all samples within groups are more similar to each other than to any sample from a different group. Software Primer 6.1.6 was used to calculate the similarity index of Sørensen and the ANOSIM analysis [55].

## Results

### Relative importance of visual and olfactory floral cues

When testing the relative importance of floral cues, we found that bees of the first and second generation responded similarly to the floral cues, irrespective of the host plants tested. In all cases, visual cues were significantly more attractive to bees when offered together with an empty cylinder control, whereas olfactory cues were not (Fig. 3a, b). When offered together with olfactory cues, visual cues were significantly more attractive to the bees. Finally, a combination of visual and olfactory cues was more attractive to bees than either cue alone.

### Comparison of attractiveness of floral cues of the different host plants

In the dual-choice bioassays testing floral cues of *T. officinale* vs. those of *C. trachelium*, we found that bees of the first and second generations did not display any preference for the cues of one host plant over the other. In all cases, decoupled visual cues of *T. officinale* and a combination of visual and olfactory cues were equally attractive to *A. bicolor* females of both generations as those equivalent cues of *C. trachelium* (Fig. 4). Responses to olfactory cues are not shown, since these were similar to responses to an empty cylinder (see above).

### Artificial flowers

The dual-choice bioassays with artificial flowers showed that yellow and lilac flowers were similarly attractive and female bees of both generations did not display a preference for a particular color (Fig. 5).

### Color analysis

The color measurements revealed that the yellow and lilac artificial flowers reflected the light in the ultra-violet (UV) color range (Fig. 6), whereas the flowers of *C. trachelium* and *T*. *officinale* did not. The flowers of *T. officinale* and the yellow artificial flowers reflected the light predominantly in the yellow, orange and red range (530–700 nm) and the flowers of *C. trachelium* and the lilac artificial flowers predominantly in the blue (380–500 nm) and red (640–700 nm) range. The colors of the host flowers and artificial flowers seemed to be distinct enough to allow discrimination by the bees. When plotting the color reflectance functions into the bee color hexagon, the color loci of flowers of *C. trachelium* and *T. officinale* and those of the yellow and lilac artificial flowers were plotted into the blue, blue-green, UV-blue and UV-green bee color spaces, respectively (Fig. 6). In the color hexagon, the pairwise distances between color loci ranged from 0.12 hexagon units (comparison between the color loci of *C. trachelium* and lilac artificial flower) to 0.53 (comparison between the color loci of *T. officinale* and lilac artificial flower). The distances between the color loci of all samples to the uncolored point (centre of the hexagon) ranged from 0.2 to 0.33 units (Additional file 3).

### Electrophysiology

**Fig. 7:**
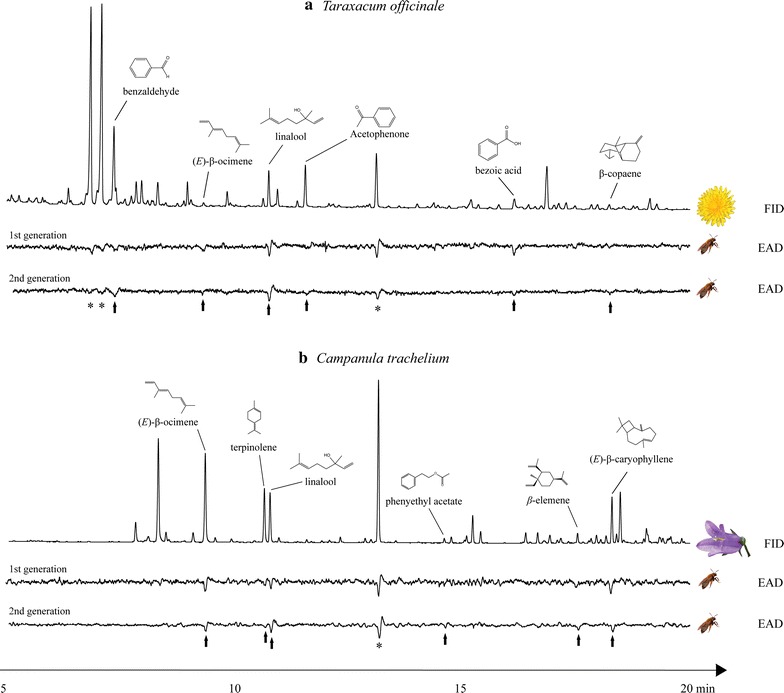
Examples of coupled gas chromatographic and electroantennographic detection (GC-EAD) of *Taraxacum officinale* (**a**) and *Campanula trachelium* (**b**) flower scent by using antennae of *Andrena bicolor* females of the first and second generation, respectively. *Asterisks* indicate compounds found in control samples (ambient contaminants)

The GC-EAD analyses with flower scent samples of *T. officinale* revealed nine electrophysiologically active compounds by using the antennae of *A. bicolo*r (Fig. 7a). The compounds triggering antennal depolarization belonged to three substance classes: aromatics (benzaldehyde, acetophenone and benzoid acid), monoterpenes [(*E*)-β-ocimene and linalool] and sesquiterpenes (β-copaene).

The GC-EAD analyses with flower scent samples of *C. trachelium* revealed seven electrophysiologically active compounds by using the antennae of *A. bicolor* (Fig. 7b). These compounds belonged to three substance classes, i.e. aromatics (phenylethyl acetate), monoterpenes [(*E*)-β-ocimene, terpinolene, linalool] and sesquiterpenes (β-elemene and (*E*)-β-caryophyllene). Only linalool and (*E*)-β-ocimene were detected in floral scent samples of both *T. officinale* and *C. trachelium*.

Antennal responses to floral scents of either *Taraxacum* (Additional file 4) or *Campanula* (Additional file 5) varied slightly among individuals. However, no significant difference was seen between the antennal responses of bees of the first and second generation. Bees of both generations responded to the same set of compounds of *T. officinale* (ANOSIM: Global *R* = −0.13; *P* = 0.93) and *C. trachelium* (ANOSIM: Global *R* = −0.15; *P* = 1).

## Discussion

The findings of our study demonstrate that *A. bicolor* females of the first and second generation have the same innate search-image, in spite of the different spectrum of host flowers that they use. In the bioassays, we found that bees of the two generations (1) have the same innate relative reliance on visual and olfactory floral cues of host plants, (2) do not prefer floral cues of *Taraxacum* to those of *Campanula* (or vice versa) and (3) do not display an innate preference for the tested colors yellow and lilac. Furthermore, in the electrophysiological analyses, bees of both generations were shown to respond within a plant species to the same set of components, all of which are ubiquitous volatiles commonly reported in plants. Together, our results suggest that the visual and olfactory constraints of *A. bicolor* loosen their innate foraging behavior, in contrast to oligolectic bees in which sensory adaptations are assumed to restrict foraging flights to specific host flowers (see below).

*Andrena bicolor* is a bivoltine solitary species that is assumed to be oligolectic on flowers of *Campanula* in the second generation but that has a broader pollen diet in the first generation [45, 46]. Based on this scenario, we have hypothesized that bees of the second generation have evolved specific sensory traits that direct their innate foraging flights towards their preferred host flowers, whereas bees of the first generation have evolved more generalized sensory traits that do not restrict their innate foraging flights to any specific host flowers. In the present study, however, various findings indicate that bees of both generations are not constrained to visit flowers of a particular host. This is in strict contrast to our hypothesis and to reports for oligolectic bees [23, 26–28, 32, 35]. First, we have found that flower-naive females of both generations do not display a remarkable preference for the floral cues of *C. trachelium* over those of *T. officinale* and vice versa. Second, in the bioassays with lilac and yellow artificial flowers, females of both generations do not show any innate preference for one color over the other. Third, in the GC-EAD analyses, bees of both generations have been determined to respond only to ubiquitous compounds that are reported as floral scent constituents of various plant species [30], in contrast to some oligolectic bees that respond additionally to highly specific volatiles of host flowers (see, for example, [32, 33, 35]). Overall, we have not detected behavioral and sensory seasonal polyphenism in *A. bicolor* and this suggests that the distinct selective forces acting on the bees of each generation are not strong enough or are acting in a similar way to keep innate foraging behavior and sensory adaptations as broad as possible in order to reduce their dependence upon a few hosts plants. Thus, irrespective of the way in which selective forces are acting on the bees of each generation, a great flexibility in innate foraging behavior seems to be an adaptive trait in *Andrena bicolor* (see below).

The absence of a clear innate preference for the floral cues of *C. trachelium* over those of *T. officinalle* and of a preference for the UV-blue bee-color of the artificial flowers typical of several *Campanula* species; [28], as well as the capability of larvae to develop on pollen of both host plants, all point to second-generation bees of *A. bicolor* being sensory and behaviorally less constrained than the oligolectic bees [26–28, 35]. The dependence of oligolectic bees on their host flowers vary considerably among species. While some species completely refuse to collect pollen in the absence of host plants [26, 27, 35], others are more flexible and may collect pollen on alternative plants if their preferred hosts are absent [12, 16, 17]. Accordingly, there might be also a great variability in sensory and behavioral constraints among these species. The summer generation of *Andrena bicolor* is known to collect pollen preferentially on flowers of *Campanula*, however, there is also report of these bees collecting pollen on alternative host plants [36] (Westrich, pers. comm]. In Germany, bees of the second generation of *A. bicolor* are active from mid June to late May–August [36, 45], whereas *Campanula* species flower predominantly between late and early August [48, 56]. Consequently, at the end of the flight season of *A. bicolor*, flowers of *Campanula* might be no longer available and a flexibility in foraging behavior by second-generation females might allow them to resume nest provisioning using alternative pollen sources.

The findings that *Andrena bicolor* does not have a particular innate preference for the floral cues of one host over the other also provide strong evidence that host-choice by adult females is not governed by conditioning with the pollen aroma during the larval stage, as hypothesized by Linsley [21]. If conditioning to pollen aroma governed the initial foraging behavior of *A. bicolor* bees, we would have expected that flower-naive adults of the first (reared on pollen of *Taraxacum*) and second (reared on pollen of *Campanula*) generation would prefer the floral cues (in this case, floral scents) of *Taraxacum* and *Campanula*, respectively, which was not the case. Consequently, in *A. bicolor*, the innate preference for a given host or the absence of it might be governed by sensory, genetically-based constraints that shape the innate foraging behavior of bees see also [10, 23].

In this study, we did not experimentally test the development of larvae of *A. bicolor* on pollen of *Taraxacum* and *Campanula* as, for example, carried out by Praz et al. [12] and Sedivy et al. [13] with other solitary bee species. Nevertheless, the observation that 170 individuals of the first generation (larvae reared mainly on *Campanula* pollen) and 190 individuals of the second generation (larvae reared mainly on *Taraxacum* pollen only) emerged in the flight cage strongly suggests that larvae of *A. bicolor* have a good capability for digesting pollen from these two hosts and probably also from other plants. Pollen of Campanulaceae and Asteraceae species vary enormously in nutritional content and digestibility [15]. Pollen of *Campanula* is protein-rich [15, 57] and is known to support the larval development of various bee species [12]. In contrast, several reports have appeared of insufficient or inappropriate larval development on the pollen of Asteraceae, including *Taraxacum officinale* (reviewed by [15]). The inability of bee larvae to develop on the pollen of Asteraceae is assumed to be related to either its low nutritional quality, e.g. low protein and amino acid contents [57–60], a difficulty in the extraction of essential compounds from the pollen grains [61], or an interference of toxic pollen compounds with nutrient digestion [12, 13]. The finding that *A. bicolor* larvae develop well on the pollen of Asteraceae, which is avoided by many other polylectic species [3], suggests that this species has physiologically evolved to deal with various pollen types, a finding with a strong implication for the evolution of pollen preference in bees (see below).

## Conclusion

The results of this study shed light not only into several aspects of the natural history of *A. bicolor*, but also into the evolution of pollen preference in bees. In a recent study, Sedivy et al. [10] proposed the persuasive constraint hypothesis of host-range evolution in bees, based mainly on findings from studies performed with oligolectic bees. According to these authors, pollen diet breadth in oligolectic bees is evolutionary constrained by sensory or neurological adaptations that might restrict their foraging flights to host flowers and by physiological adaptations that might restrict their capability to digest different pollen types. However, evidence that polylectic bees are adapted sensory and physiologically to exploit a broad spectrum of host flowers is scarce. In this study, we have found, for the first time, evidence that polylectic solitary bees are visually and olfactory less constrained than the oligolectic bees investigated so far and that this might allow them to visit a broad spectrum of flowers. Furthermore, we have revealed that *A. bicolor* has a broad digestive capability that allows its larvae to develop even on Asteraceae pollen, which is difficult to digest for several other polylectic species [3]. The different levels of sensory and physiological constraints observed in bees might be directly related to their ability to exploit pollen from either a few or several host plants and might help to explain the continuum of pollen preference (from monolecty to broad polylecty) observed in bees. Although our understanding about the mechanisms governing the evolution of host-choice in bees has improved considerably in the last decade, the conclusions traced so far are based on a very few species. Thus, more effort is still necessary to establish to what extent sensory, neurological and physiological adaptations shape the evolution of host preference in bees.

## Additional files

10.1186/s12898-016-0074-z Detailed procedures applied for color measurements, bee color hexagon, sampling of floral scents, electrophysiology and chemical analyses.
10.1186/s12898-016-0074-z Proportion of approach (AP) and landing (LA) responses of *Andrena bicolor* females to floral cues of *Taraxacum officinale* and *Campanula trachelium*.
10.1186/s12898-016-0074-z Euclidean distances (in hexagon units) among color loci of flowers of *C. trachelium* and *T. officinale* and of lilac and yellow artificial flowers.
10.1186/s12898-016-0074-z Electrophysiological responses of bees of the first and second generation of *Andrena bicolor* to floral scents of *Taraxacum*.
10.1186/s12898-016-0074-z Electrophysiological responses of bees of the first and second generation of *Andrena bicolor* to floral scents of *Campanula*.

## Abbreviations

GC/EAD: gas chromatography coupled with electroantennographic detection
GC/MS: gas chromatography coupled with mass spectrometry
