# Supplementary material for: Host choice in a bivoltine bee: how sensory constraints shape innate foraging behaviors
Source: BMC Ecol. 2016 Apr 11;16:20. doi: 10.1186/s12898-016-0074-z (PMC4828851; doi:10.1186/s12898-016-0074-z)
Supplement: Supplementary file 1 — 10.1186/s12898-016-0074-z Detailed procedures applied for color measurements, bee color hexagon, sampling of floral scents, electrophysiology and chemical analyses. [file 12898_2016_74_MOESM1_ESM.docx]

**Host choice in a bivoltine bee: how neurological constraints shape innate foraging behaviors**

Paulo Milet-Pinheiro^1^*^#^, Kerstin Herz^1^, Stefan Dötterl^2^, Manfred Ayasse^1^

* Corresponding Author: Paulo Milet-Pinheiro (miletpinheiro@hotmail.com)

## **Additional File 1**

# Material and Methods

## **Colour measurements and bee-colour hexagon**

The spectral reflection properties of the corolla of *T. officinale* and *C. trachelium* and of yellow and blue artificial flowers were recorded from 300 to 700 nm [[the wavelength perceived by bees; 1](#_ENREF_1)] by using a Varian Cary 5 spectrophotometer (Varian, Inc, Palo Alto, California). We measured the corolla of three plant individuals per species (*n* = 1 flower per individual) and three different regions of the paper used for fashioning the blue and yellow artificial flowers. Barium sulfate and an open black film canister were used as the white and black references, respectively.

The mean reflections of both petals and paper (comprising the three replicates measured for each) were used to determine the colour loci in the hexagon colour space [[2](#_ENREF_2)]. We applied the daylight irradiance spectrum D65 as a standard and used the spectral sensitivity of honeybee’s photoreceptors [[3](#_ENREF_3)]. Given that bees do not differ substantially in their visual sensory systems [[1](#_ENREF_1)], we used the spectral sensitivity functions described for honeybees as a representative approximation for *A. bicolor* [[3](#_ENREF_3)]. The position of the colour loci show the way that bees perceive the corolla through their ultraviolet, blue and green photoreceptors and through further processing of receptor signals in the central nervous system [[4](#_ENREF_4)]. The colour hexagon is separated into six different colour sectors representing the different bee colour spaces, i.e. UV, UV-blue, blue, blue-green, green, UV-green [[5](#_ENREF_5)].

For a comparison of the bee colours among the flowers of *T. officinale* and *C. trachelium* and of those of the artificial flowers, the pairwise hexagon distances of the colour loci among species and the distance of each colour locus to its background (green leaves) were calculated [[3](#_ENREF_3)]. The reflectance function of a typical green leaf was used as the background colour [[5](#_ENREF_5)]. Behavioural experiments with bumblebees trained to visit artificial flowers have demonstrated that colour distances of 0.05 hexagon units are poorly discriminated, whereas distances greater than 0.10 are easily discriminated [[6](#_ENREF_6), [7](#_ENREF_7)].

## **Sampling of floral scent**

Floral volatiles from *T. officinale* and *C. trachelium* were sampled by using standard dynamic headspace methods. From each species, we collected five samples that were pooled afterwards for electrophysiological analyses (see below). For each sample, ten flowers each from different individuals were cut at the base of the calyx and placed into a clean glass chamber (5 cm in height and 9 cm in diameter) with two openings. The chamber was closed with a polyester oven bag (Toppits®). To reduce the contamination of the samples by the volatiles released after injury, "tissue bleeding" was removed from the cut with a filter paper. Simultaneously to each flower scent sample and by using the same methods, we collected control samples that consisted of a glass chamber filled with ten pieces (2 cm long) of either pedicel (as a control for *T. officinalle*) or stem (*C. trachelium*). The volatiles within the chambers were trapped for 4 h in an adsorbent tube through which air was drawn at a rate of 100 ml/min by using a membrane pump (G12/01 EB, Rietschle Thomas, Puchheim, Germany). The incoming air stream was cleaned of ambient contaminants by a charcoal filter (50 mg, Supelco, Orbo32 small). The adsorbent tubes consisted of glass tubing (length 9 cm; inner diameter 2 mm) filled with a mixture of 5 mg Tenax (mesh 60/80; Supelco) and 5 mg Carbotrap (mesh 20/40; Supelco) fixed within the tubing with glass wool (silane-treated; Supelco). The volatiles retained in the adsorbent tubes were then eluted with 300 µl n‑hexane (98%; Merck). The headspace samples were stored in screw cap vials at −20 °C until electrophysiological and chemical analyses.

## **Electrophysiology**

Electroantennographic detection coupled with gas chromatography (GC/EAD) was conducted on *A. bicolor* females of both generations to determine the antennal perception of the bee for compounds in the floral scent bouquet of both *T. officinale* and *C. trachelium*. The GC-EAD system consisted of a HP 6890 gas chromatograph (Agilent Technologies, Germany) equipped with an FID and a non-polar DB-5 column (30 m x 0.25 mm; film thickness 0.25 µm; J&W; USA) and coupled to an EAD setup (Syntech, Hilversum; Netherlands). Aliquots (1 µl) of the headspace samples of *T. officinale* and of *C. trachelium* were injected splitless into the GC injector at an initial temperature of 50 °C. The oven temperature was increased at a rate of 6 °C/min to a final temperature of 220 °C, which was held for 5 min. The GC effluent was split (split ratio FID:EAD = 1:1) by using a four-arm splitter (GRAPHPACK 3D/2, Gerstel, Mülheim, Germany) and 30 ml/min of make-up gas (nitrogen) was added. The outlet of the EAD was placed in a cleaned and humidified constant airflow (100 ml/min) that was directed over the antennal preparation.

Antennae of *A. bicolor* females (*n* = 5 for each generation) were cut at the base and the tip and mounted between two glass capillaries connected to gold electrodes, thereby closing an electric circuit. The capillaries were filled with insect Ringer solution (5 g NaCl; 0.42 g KCl; 0.19 g CaCl₂ in 1000 ml demineralised water). A scent compound was considered to be EAD-active when it elicited a depolarization response in at least three of the five replicates in each generation.

## **Chemical Analyses**

To identify the volatiles eliciting antennal depolarization in *A. bicolor*, the headspace samples of *T. officinale* and *C. trachelium* used for the electrophysiological measurements were analyzed on a Shimadzu GC-MS-QP2010 Ultra equipped with a AOC-20i autoinjector (Shimadzu, Tokyo, Japan) and a fused silica column ZB-5 (5% phenyl polysiloxane; 60 m long, inner diameter 0.25 mm, film thickness 0.25 µm, Phenomenex). Injection temperature was 26°C (split ratio 1:3) and the column flow (carrier gas: helium) was set at 250 °C and 1.5 ml/min, respectively. The GC oven temperature was held for 1 min at 40 °C and then increased by 6 °C per minute to 250 °C. The MS interface worked at 260 °C and the ion source at 200 °C. Mass spectra were taken at 70 eV (in EI mode) from m/z 30 to 350. The GC/MS data were processed by using the GCMSolution Version 2.72 package.

Tentative identification of most of the compounds was carried out by using the NIST 08, Wiley 7, and Adams (Adams 2007) mass spectral data bases or the data base provided in MassFinder 3 and was confirmed by a comparison of retention times with published data. Structural assignments of individual components were confirmed by a comparison of mass spectra and GC retention times with those of authentic standards.

**References**

1. Peitsch D, Fietz A, Hertel H, Souza J, Ventura DF, Menzel R. The Spectral Input Systems of Hymenopteran Insects and Their Receptor-Based Colour Vision. J Comp Physiol A Sens Neural Behav Physiol. 1992;170:23-40.

2. Chittka L. The colour hexagon: a chromaticity diagram based on photoreceptor excitations as a generalized representation of colour opponency. J Comp Physiol A. 1992;170:533-543.

3. Chittka L, Kevan PG. Flower colour as advertisement. In: Dafni A, Kevan PG, Husband BC, editors. Practical Pollination Biology. Cambridge: Enviroquest Ltd; 2005. p. 157 - 196.

4. Raine NE, Chittka L. The adaptive significance of sensory bias in a foraging context: floral colour preferences in the bumblebee *Bombus terrestris*. Plos One. 2007;556:1-8.

5. Chittka L, Shmida A, Troje N, Menzel R. Ultraviolet as a componet of flowers reflections, and the colour perception of hymenoptera. Vision Res. 1994;34:1489-1508.

6. Dyer AG, Chittka L. Biological significance of distinguishing between similar colours in spectrally variable illumination: bumblebees (*Bombus terrestris*) as a case study. J Comp Physiol A. 2004;190:105-114.

7. Dyer AG, Chittka L. Fine colour discrimination requires differential conditioning in bumblebees. Naturwissenschaften. 2004;91:224-227.
