# Supplementary material for: Host choice in a bivoltine bee: how sensory constraints shape innate foraging behaviors
Source: BMC Ecol. 2016 Apr 11;16:20. doi: 10.1186/s12898-016-0074-z (PMC4828851; doi:10.1186/s12898-016-0074-z)
Supplement: Supplementary file 2 — 10.1186/s12898-016-0074-z Proportion of approach (AP) and landing (LA) responses of Andrena bicolor females to floral cues of Taraxacum officinale and Campanula trachelium. [file 12898_2016_74_MOESM2_ESM.doc]

**Host choice in a bivoltine bee: how neurological constraints shape innate foraging behaviors**

Paulo Milet-Pinheiro^1^*^#^, Kerstin Herz^1^, Stefan Dötterl^2^, Manfred Ayasse^1^

* Corresponding Author: Paulo Milet-Pinheiro (miletpinheiro@hotmail.com)

## Additional file 2

Proportion of approach (AP) and landing (LA) responses of *Andrena bicolor* females to floral cues of *Taraxacum officinale* and *Campanula trachelium*. Fisher's exact tests were used to test whether decoupled visual cues of flowers of *T. officinale* and *C. trachelium* and a combination of visual and olfactory cues triggered approach and landing responses in bees in a similar proportion.

| Floral cues | 1st generation bees | | |  | 2nd generation bees | | |
| --- | --- | --- | --- | --- | --- | --- | --- |
|  | AP | LA | (p) |  | AP | LA | (p) |
| Visual *Taraxacum* | 7 | 6 | 1 |  | 18 | 13 | 0.79 |
| Visual *Campanuala* | 8 | 6 |  |  | 13 | 11 |  |
|  |  |  |  |  |  |  |  |
| Visual + olfactory *Taraxacum* | 10 | 9 | 1 |  | 20 | 17 | 1 |
| Visual + olfactory *Campanula* | 12 | 12 |  |  | 15 | 13 |  |
