# Supplementary material for: Host choice in a bivoltine bee: how sensory constraints shape innate foraging behaviors
Source: BMC Ecol. 2016 Apr 11;16:20. doi: 10.1186/s12898-016-0074-z (PMC4828851; doi:10.1186/s12898-016-0074-z)
Supplement: Supplementary file 3 — 10.1186/s12898-016-0074-z Euclidean distances (in hexagon units) among color loci of flowers of C. trachelium and T. officinale and of lilac and yellow artificial flowers. [file 12898_2016_74_MOESM3_ESM.docx]

**Host choice in a bivoltine bee: how neurological constraints shape innate foraging behaviors**

Paulo Milet-Pinheiro^1^*^#^, Kerstin Herz^1^, Stefan Dötterl^2^, Manfred Ayasse^1^

* Corresponding Author: Paulo Milet-Pinheiro (miletpinheiro@hotmail.com)

## Additional file 3

Euclidean distances (in hexagon units) among color loci of flowers of *C. trachelium* and *T. officinale* and of lilac and yellow artificial flowers. Distances to the hexagon centre are also given.

|  | *C. trachelium* | *T. officinale* | Lilac | Yellow | Centre |
| --- | --- | --- | --- | --- | --- |
| *C. trachelium* |  | 0.44 | 0.12 | 0.45 | 0.20 |
| *T. officinale* |  |  | 0.53 | 0.35 | 0.33 |
| Lilac |  |  |  | 0.47 | 0.24 |
| Yellow |  |  |  |  | 0.25 |
