# Supplementary material for: Host choice in a bivoltine bee: how sensory constraints shape innate foraging behaviors
Source: BMC Ecol. 2016 Apr 11;16:20. doi: 10.1186/s12898-016-0074-z (PMC4828851; doi:10.1186/s12898-016-0074-z)
Supplement: Supplementary file 5 — 10.1186/s12898-016-0074-z Electrophysiological responses of bees of the first and second generation of Andrena bicolor to floral scents of Campanula. [file 12898_2016_74_MOESM5_ESM.doc]

**Host choice in a bivoltine bee: how neurological constraints shape innate foraging behaviors**

Paulo Milet-Pinheiro^1^*^#^, Kerstin Herz^1^, Stefan Dötterl^2^, Manfred Ayasse^1^

* Corresponding Author: Paulo Milet-Pinheiro (miletpinheiro@hotmail.com)

**Additional file 5**

Antennal responses of first and second generation females of *Andrena bicolor* to floral scent compounds of *Campanula trachelium*. Only compounds considered to be EAD-active, i.e. those compounds triggering antennal responses in at least three of five individuals tested for each generation, are listed. "1" and "0" represent presence or absence of responses, respectively.

|  |  | **1st generation bees** | | | | |  | **2nd generation bees** | | | | |
| --- | --- | --- | --- | --- | --- | --- | --- | --- | --- | --- | --- | --- |
| **Compounds** |  | A | B | C | D | E |  | A | B | C | D | E |
| (*E*)-β-ocimene |  | 1 | 1 | 1 | 1 | 1 |  | 1 | 1 | 1 | 1 | 1 |
| Terpinolene |  | 1 | 1 | 1 | 0 | 0 |  | 1 | 1 | 1 | 0 | 1 |
| Linalool |  | 1 | 1 | 1 | 1 | 1 |  | 1 | 1 | 1 | 1 | 1 |
| Phenylethyl acetate |  | 0 | 1 | 1 | 1 | 1 |  | 1 | 1 | 1 | 1 | 0 |
| β-elemene |  | 1 | 0 | 0 | 1 | 1 |  | 1 | 1 | 0 | 1 | 1 |
| (*E*)-β-caryophyllene |  | 1 | 1 | 1 | 1 | 1 |  | 1 | 1 | 1 | 1 | 1 |
